# Disclosing pathogenic processes of monkeypox (MPOX) and drug repurposing through host-transcriptomics analysis

**DOI:** 10.1371/journal.pntd.0014797

**Published:** 2026-10-16

**Authors:** Sabkat Mahmud, Alvira Ajadee, Md. Ahad Ali, Md. Feroj Ahmed, Reaz Ahmmed, Md. Masud Rana, Tasfia Noor, Md. Fahim Faysal, Md. Hadiul Kabir, Md. Nurul Haque Mollah

**Affiliations:** 1 Bioinformatics Lab (Dry), Department of Statistics, University of Rajshahi, Rajshahi, Bangladesh; 2 Tulane Center for Biomedical Informatics and Genomics, Deming Department of Medicine, School of Medicine, Tulane University, New Orleans, Louisiana, United States of America; 3 Department of Chemistry and Biochemistry, University of Texas at El Paso, El Paso, Texas, United States of America; 4 Department of Biochemistry & Molecular Biology, University of Rajshahi, Rajshahi, Bangladesh; 5 Department of Statistics and Data Science, University of Barishal, Barishal, Bangladesh; 6 Department of Computer Science and Engineering, Rajshahi University of Engineering and Technology, Rajshahi, Bangladesh; Australian Red Cross Lifeblood, AUSTRALIA

## Abstract

Monkeypox (MPOX) virus infection outbreak in Africa in 2022 was initially considered a neglected tropical disease (NTD). Now it is recognized as a global health problem. The recent global spread has emphasized the need for increased attention to combat MPOX. Although there are some vaccines against MPOX, most people worldwide are still not vaccinated due to their unawareness or unavailability. However, so far, there is no approved drug against MPOX yet. Therefore, effective drugs against MPOX are also essential. This study attempted to disclose pathogenic processes and therapeutic strategies of MPOX based on MPOX-associated host key-genes (hKGs). At first, three transcriptomic profile datasets were analyzed, identifying 166 common host differentially expressed genes (hDEGs) between MPOX patients and control groups, comprising 75 upregulated and 91 downregulated hDEGs. Subsequently, six top-ranked host key genes (hKGs): ALB, HSP90AA1, CDH1, PTPRC, IGF1, and ABL1, were identified as potential MPOX-causing genes through protein-protein interaction (PPI) network analysis. Functional enrichment analysis of these hKGs revealed critical pathogenic processes of MPOX infection, including biological processes, molecular functions, cellular components, and KEGG pathways. Regulatory network analysis involving transcription factors and miRNAs provided further insights into disease mechanisms. Based on molecular docking analysis, five top-ranked drug molecules Tecovirimat, Beclabuvir, Maraviroc, Raltegravir, and Brincidofovir were identified as potential repurposable therapeutics against MPOX. ADME/T and drug-likeness evaluations confirmed favorable pharmacokinetic properties for these candidates. Additionally, 100 ns molecular dynamics (MD) simulations of the top three complexes (ALB-Tecovirimat, CDH1-Maraviroc, and PPARG-Beclabuvir) demonstrated strong binding stability, supported by RMSD, RMSF, and MM-GBSA results. Therefore, the results provided in this study might be potential resources for diagnosis and therapies of MPOX patients. However, the findings require experimental validation in wet lab for further confirmation.

## Author summary

Monkeypox virus infection has become a one of the global health concerns, after COVID-19. Although vaccines are available, many people still have limited access to the vaccination, and so effective treatments are needed. In antiviral drug discovery, potential targets may include pathogenic viral proteins or host proteins that are dysregulated by the interaction of pathogenic viral proteins. However, viral proteins guided antiviral drugs may become inactive/resistant, since virus RNAs are often modified due to environmental stress. In contrast, host proteins tend to be more stable and less susceptible for such situations, making them attractive alternative targets for antiviral strategies. We therefore identified host key-genes associated with the Monkeypox virus infection through transcriptomics analysis for exploring effective antiviral candidates. We then investigated biological processes, molecular functions, cellular components and pathways that might be associated with infection through the enrichment analysis of key-genes. Finally, key-genes guided five molecules were recommended as the. drug candidate against the infection. However, these findings are based on computational analyses, so laboratory experiments and clinical studies will be necessary to finalize them.

## 1 Introduction

Monkeypox (MPOX) virus infection is caused by a double-stranded DNA virus and has long been a public health threat. The outbreak of MPOX in May 2022 has significantly worsened social life, for which the World Health Organization (WHO) declared it as a Public Health Emergency of International Concern (PHEIC) on July 23, 2022 [1]. After COVID-19, this is the major threat as a PHEIC [2]. This infection was first identified in 1958, and the first human case was documented in 1970 in the Democratic Republic of Congo. It is endemic to West and Central African countries, particularly in regions with close contact between humans and wildlife [3]. Recent outbreaks of MPOX in non-endemic countries have drawn significant attention due to their potential for widespread transmission, emphasizing the need for enhanced global surveillance and coordinated public health responses [4]. The MPOX virus exists in hosts in two distinct forms: intracellular mature virus (MV) and extracellular enveloped virus (EV). MV enters into host cells through attachment and fusion/lysis to release genetic materials, while EV exits through exocytosis [5]. It evades immune detection by inhibiting interferon signaling and modulating antiviral mechanisms, which allows the virus to persist [6]. The virus interacts with host cells by binding with surface receptor proteins to enter into the cells via endocytosis and hijacks the host’s transcription machinery, and the modified virus replicates in the cytoplasm and develops infection by dysregulating the receptor proteins. A tentative mechanism of MPOX infection might be illustrated as in Fig 1, where the necessary data for this figure was collected from previous studies [7,8]. This infection triggers an immune response, involving both innate and adaptive immunity, leading to symptoms such as fever, rash, headache, and lymphadenopathy [9–11]. Severe infections may cause complications like secondary infections and respiratory distress [6]. However, researchers’ understanding on its pathogenic processes and therapeutic strategies are not yet reach to the satisfactory level.

**Fig 1:**
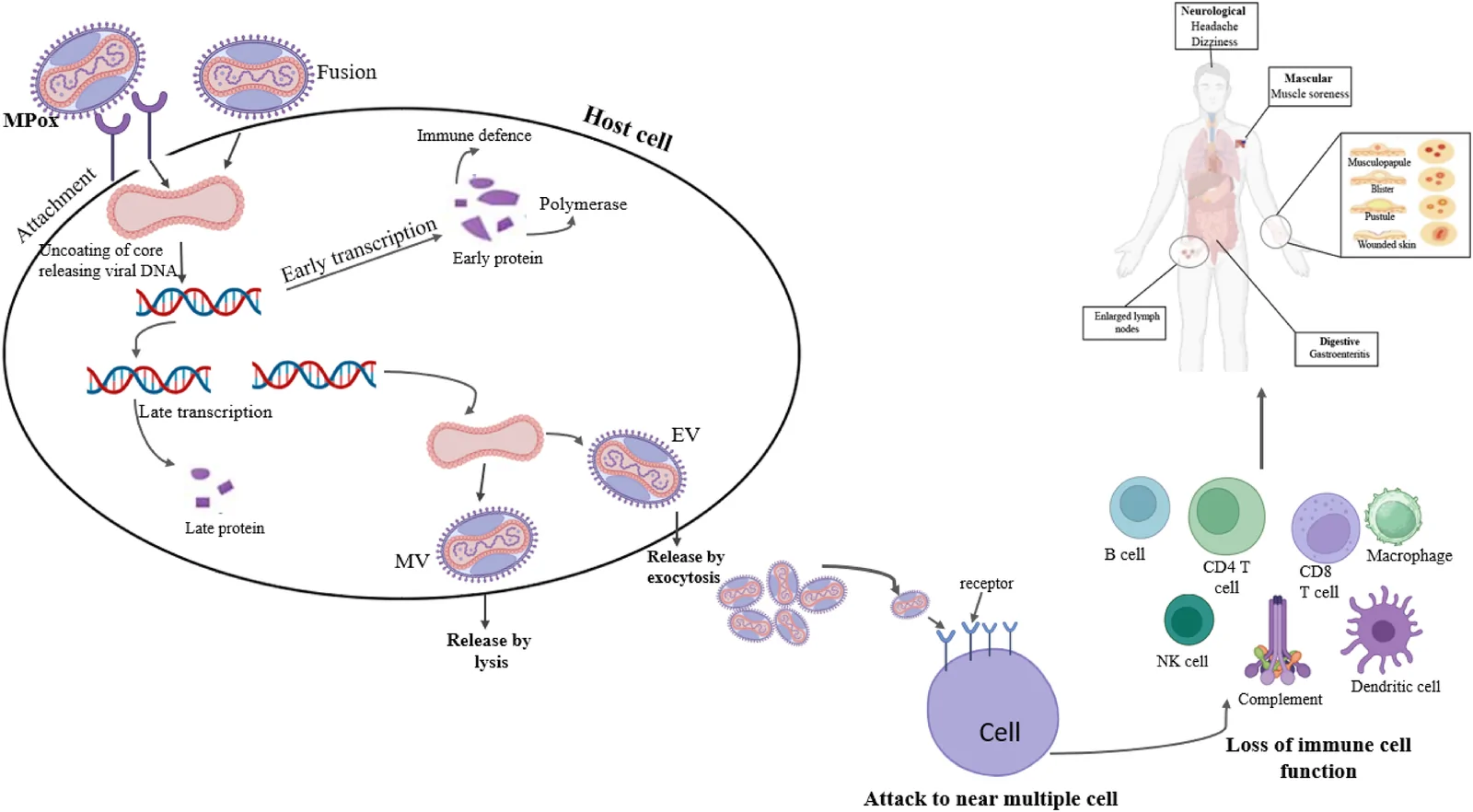
A schematic diagram of MPOX infection mechanism.

There are some vaccines, including JYNNEOS and ACAM2000, against MPOX, but majority of the people worldwide are still not vaccinated due to their unawareness or unavailability of the vaccines [12–14]. Although several oral antivirals, including Tecovirimat (TPOXX, ST-246), which is FDA-approved for the treatment of smallpox and has shown activity against orthopoxviruses, Brincidofovir (CMX001, Tembexa), which is also FDA-approved and has been evaluated for orthopoxvirus infections, and cidofovir has been used off-label in selected severe MPOX cases, but their clinical effectiveness remains limited [15–17]. Therefore, effective drugs against MPOX are essential. In antiviral drug discovery, potential drug targets may include viral proteins essential for viral infection, replication, and pathogenesis, as well as host proteins and cellular pathways that are hijacked, modulated, or dysregulated by viral proteins [18–23], since viral proteins interact with host proteins and cellular machinery to facilitate infection and viral replication [24–29]. However, viral proteins guided antiviral drugs may become inactive/resistant against the targeted viral proteins [30,31], since virus RNAs are often modified due to environmental stress. In contrast, host-targeted proteins tend to be more stable and less susceptible to such variations, making them attractive alternatives for antiviral strategies [32,33]. To overcome the problem of anti-viral drug-resistance, it may be required to explore infection-causing host genes/proteins-guided drugs against MPOX. There are two ways (repurposing and de-novo) of exploring candidate drug molecules. But de-novo approach is laborious, time consuming and costly compare to the repurposing approach. The drug repurposing strategy explores existing drugs of different diseases for their new application in other diseases of interest. Therefore, specific objectives of this study are to disclose pathogenic processes of monkeypox (MPOX) and repurposable candidate drugs through host-transcriptomics analysis using bioinformatics approaches.

## 2 Methodology

To explore repurposable drugs for MPOX, it is required to identify MPOX-causing hKGs as the targets of potential drug molecules. Transcriptomic profile analysis through bioinformatics tools is a popular approach to detect disease-causing hKGs as drug targets [34–37].

### 2.1 Data sources and descriptions

#### 2.1.1 Transcriptomics profiles (case/control) acquisition.

To investigate the transcriptional impact of MPOX virus infection on human cells, we analyzed three microarray datasets: GSE36854 (2 MPOX-infected and 2 control samples), GSE24125 (24 MPOX-infected and 28 control samples), and GSE11234 (48 MPOX-infected and 28 control samples) retrieved from the GEO database, sequenced on the GPL4133, GPL10912, and GPL6762 platforms, respectively. The detail information about the datasets can be found in S1 Table. We considered only MPOX-infected samples as a case in these three datasets.

#### 2.1.2 Collection of candidate drug molecules.

To explore potential drug candidates for MPOX treatment, we collected 215 antiviral drug molecules through systematic literature review related to MPOX disease and used the DrugBank database [38] to retrieve the drug information (S2 Table). In addition, we used DGIdb database [39] to collect the gene targeted drug molecules and finally we identified 100 hKGs-guided drugs. By integrating these resources, we aim to identify promising candidates for drug repurposing to mitigate the effects of MPOX infection.

### 2.2 Identification of host DEGs (hDEGs) from transcriptomics profiles

We considered LIMMA r-package [40] to explore differentially expressed genes (DEGs) between MPOX and control groups, since LIMMA works well for both small and large sample sizes in the case of microarray gene-expression data [41]. We computed adj. *p*-values using Benjamini–Hochberg (BH) procedure in LIMMA approach and the average of log_2_ fold-change (aLog_2_FC) values with each gene to test the null hypotheses (H_O_): similar expression patterns in both MPOX and control groups against the alternative hypotheses (H1): differential expression patterns between two MPOX and control groups. Under this hypothesis we found three DEGs sets for three datasets with NCBI accession IDs GSE36854, GSE24125 and GSE11234, respectively. The scores of aLog_2_FC were used to distinguish upregulated and downregulated DEGs as follows,


 DEGg={Up(DEG),if adj.p.value≤0.05 and aLog2 FCg≥+1.0 Down(DEG),if adj.p.value≤0.05 and aLog2 FCg≤−1.0

where,


$$\begin{array}{c} aLog_{2}FC_{g}=\left\{ \begin{array}{c} \frac{1}{n_{1}}\sum_{i}^{n_{1}}\log_{2}\;\left(z_{gi}^{T}\right)-\frac{1}{n_{2}}\sum_{j}^{n_{2}}\log_{2}\;\left(z_{gj}^{C}\right),\;\text{if}\;n_{1}\neq n_{2},\\ {}[8pt]\frac{1}{n}\sum_{i}\log_{2}\;\left(\frac{z_{gi}^{T}}{z_{gj}^{C}}\right),\;\text{if}\;n_{1}=n_{2}=n \end{array}\right. \end{array}$$

Here $z_{gi}^{T}$ and $z_{gj}^{C}$ are the responses/expressions for the *g*th gene with the *i*th case and *j*th control samples, respectively. To avoid the influence of batch effects and platform heterogeneity, DEGs were detected from each of three datasets separately. LIMMA applies empirical Bayes–moderated statistics to stabilize gene-wise variance estimates by borrowin*g* information across all genes, thereby improving statistical power and reliability, particularly in datasets with limited sample sizes [35,42]. Only genes that were consistently and significantly detected as DEGs with each dataset were considered as common host differentially expressed genes (chDEGs).

### 2.3 MPOX-causing host Key Genes (hKGs) Identification from hDEGs

Proteins carry out their functions by interacting with other proteins in the cell and by producing data that improves our understanding of the protein’s function through the protein-protein interaction (PPI) network [43]. To explore hKGs, an online database and analysis tool (STRING v11.5) was used with its default parameters to create the PPI network of hDEGs. The network was visualized using the Cytoscape software [44]. The CytoHubba [45] plugin in Cytoscape was used to select hKGs based on different centrality measures (Degree, Closeness, EPC, MNC, Radialty, and MCC). These measures allowed us to identify nodes with critical roles in the network topology. At first, top eight hKGs were selected based on the top-rank genes across the centrality measures. Then the stability of these hKGs were evaluated through bootstrap resampling of PPI network for 100 replications implemented by the Python NetworkX library [46]. In each replication, this approach computed three centrality measures (degree, closeness, and betweenness) independently. The hKGs that appeared as the members of top eight genes in at least 80 out of 100 replications based on at least two centrality measures, were considered as the stable hKGs for further investigations.

### 2.4 Identification of key regulators for hKGs

We conducted interaction network analysis of hKGs with the transcription factors (TFs) and micro RNAs (miRNA) to explore key transcriptional and post-transcriptional regulators of hKGs. In order to determine transcriptional and post-transcriptional regulators, we created TFs-hKGs interaction network with the JASPAR database [47], and miRNAs-hKGs interaction network with the TarBase [48] database, respectively. NetworkAnalyst [49] was used to compute these interactions. Both transcriptional and post-transcriptional regulators were selected using two topological centrality measures degree and betweenness. Higher centrality scores in both degree and betweenness measures often indicate stronger regulators hKGs, as they capture complementary aspects of influence. Degree highlights hubs with many direct targets, while betweenness identifies bottlenecks controlling information flow between hKGs [50]. We used Cytoscape [51] to visualize the networks of their interactions.

### 2.5 The hKGs-set enrichment analysis with GO-terms and KEGG-pathways

The Gene Ontology (GO) project is a key bioinformatics resource that offers comprehensive functional data on gene products and their biological activities [52]. To investigate the functional roles and pathways associated with hKGs, we performed hKGs-set enrichment analysis using GO terms and KEGG pathways. This analysis was conducted using the GeneCodis database [53], applying a Fisher’s exact test with a cutoff at a *p*-value of 0.05 to identify statistically significant enrichments. This approach allowed us to explore the biological activities and pathways in which these hKGs are involved, highlighting their potential roles in disease processes.

### 2.6 The hKGs-guided drug repurposing

To explore hKGs-guided repurposable drug molecules for MPOX infection, we conducted molecular docking to evaluate binding affinities with hKGs. We assessed drug-likeness properties, pharmacokinetics, and toxicity, and performed dynamic simulations to ensure the efficacy, safety, and stability of candidate drug molecules as discussed in subsections 2.6.1-2.6.4.

#### 2.6.1 Exploring candidate drugs by molecular docking.

Two primary *in-silico* approaches for drug discovery are *de-novo* design and drug repurposing. *De-novo* design is often time-consuming and costly, whereas drug repurposing leverages already approved or clinically investigated drugs for new therapeutic applications. In both strategies, molecular docking is commonly used as an exploratory virtual screening tool to prioritize candidate molecules by predicting their binding affinities and interaction modes with target proteins, rather than to establish definitive therapeutic efficacy [34,35,42,54,55]. To explore repurposable drugs for MPOX, molecular docking was performed using AutoDock Vina (*v1.5.6*) [56] which is widely recognized for its accuracy and computational efficiency. Protein structures were prepared by removing water molecules, adding polar hydrogens, and assigning Kollman charges, while ligand structures were optimized using the MMFF94 force field. Docking grid dimensions were defined to encompass the entire binding pocket to allow sufficient ligand flexibility, and the exhaustiveness parameter was set to 8 to improve search accuracy. Host key genes (hKGs) and their associated top transcription factor proteins were considered as target receptors. A total of 315 candidate drug molecules were collected from online databases (S2 Table). Three-dimensional structures of receptor proteins were obtained from the Protein Data Bank [57] and AlphaFold databases [58]. The 3D structures of all 315 MPOX-related meta-drug candidates were retrieved from the PubChem database [59]. Binding affinity scores (in kcal/mol) between receptors and ligands were calculated using AutoDock Vina. Receptor proteins and drug molecules were ranked based on the ascending order of their average binding affinity scores. A binding affinity scores (BAS) <-7.0 kcal/mol was applied, as the cutoff which is commonly used in the structure-based drug discovery to indicate moderate-to-strong binding interactions [60,61]. In this study, we hypothesized that drug candidates exhibiting a BAS of ≤ -7.0 kcal/mol toward their respective target proteins are more likely to effectively bind and modulate the target, whereas compounds with BAS values > -7.0 kcal/mol are less likely to exhibit favorable target interactions. Nevertheless, docking scores alone do not confirm biological activity and should be interpreted in conjunction with pharmacokinetic analyses and experimental validation.

#### 2.6.2 Drug-likeness properties.

Evaluating the drug-likeness properties of candidate drug molecules is essential for checking their effective bioavailability and safety. This assessment helps ensure that the molecule possesses the necessary characteristics for successful drug development and clinical application, thereby increasing their likelihood of success in treating target diseases. Drug-likeness of the selected compounds was assessed based on Lipinski’s Rule of Five [62]. Physicochemical and medicinal chemistry properties were predicted using ADMETlab 2.0 [63] and pkCSM [64] including parameters such as molecular weight, hydrogen bonding, logP, PAINS alerts, Brenk filters, and synthetic accessibility. SMILES representations of the compounds were obtained from the PubChem database [65].

#### 2.6.3 Pharmacokinetics (PK) and Toxicity analysis.

Pharmacokinetics (ADME) describes how a compound behaves in the body after administration. In drug repurposing, strong binding alone is not sufficient, as poor absorption, rapid elimination, or toxicity can limit clinical usefulness. Therefore, ADME/T evaluation is essential to screen and prioritize compounds with suitable safety and pharmacokinetic profiles before further investigation [66–69]. The ADMETlab 2.0 [63] web server was used to predict the ADME features of the tested drug molecules. Additionally, toxicity properties were investigated using ProTox [70] and pkCSM [64] web tools, which assess pharmacokinetics and potential toxicity in the human body. Key organ toxicity properties such as hERG inhibition, carcinogenicity, maximum tolerated dose, acute toxicity, AMES toxicity, and skin sensitization were evaluated using pkCSM web tools to ensure the safety of the selected drug molecules.

#### 2.6.4 Connectivity Map analysis of prioritized drug candidates.

To provide computational validation of the prioritized therapeutic candidates, Connectivity Map (CMap) analysis was performed using the CMap and LINCS Unified Environment [71,72]. The significantly upregulated and downregulated host genes identified from the differential gene expression analysis were used as the disease transcriptional signature. Separate gene lists containing upregulated and downregulated genes were submitted to the CLUE query interface. The platform compares the submitted disease signature against a large collection of drug-induced gene expression signatures generated by the Library of Integrated Network-based Cellular Signatures (LINCS) L1000 project using a rank-based enrichment algorithm [71,72]. For each perturbagen, CLUE computes a Normalized Connectivity Score (NCS), which quantifies the similarity or opposition between the disease-associated and drug-induced transcriptional signatures. Compounds with negative NCS values indicate transcriptional profiles that oppose the disease signature and were therefore considered potential candidates capable of reversing disease-associated molecular perturbations [72]. Candidate compounds exhibiting consistently negative connectivity scores across multiple human cell lines were prioritized as computationally validated therapeutic candidates.

#### 2.6.5 Molecular dynamics (MD) simulation.

MD simulations were used to confirm the molecular docking results. These simulations help evaluate the stability of protein-ligand complexes by providing insights into fluctuations and conformational changes as the system stabilizes [73]. The simulations were run in a periodic water box for 100 nanoseconds using the OPLS3e force field and Desmond v2020-4 (Schrödinger, Academic) on Linux environment. The protein-ligand complexes were positioned in a rectangular box with a 10 Å buffer, filled with SPC (Simple Point Charge) water model. The systems were initially neutralized by adding appropriate counterions to eliminate net charge, followed by the addition of Na⁺ and Cl^-^ions to reach a physiological ionic strength of 0.15 M. After system construction, energy minimization was performed to relieve steric clashes and unfavorable contacts. The systems were then gradually equilibrated under restrained NVT conditions to 300 K, followed by NPT equilibration at 300 K and 1 atm to stabilize density and pressure prior to production simulations [74]. The Lincs method was used to constrain hydrogen bonds, with a 2-fs time step. Van der Waals interactions were evaluated with a switching range of 12–14 Å and a cutoff of 14 Å. Electrostatic interactions were calculated with the Particle Mesh Ewald (PME) method, with a grid spacing of 1.2 Å [75]. After 100 ns of energy minimization, the resulting trajectory was used for MD simulations. These simulations were performed for 100 nanoseconds (ns) with a time step of 2 femtoseconds (fs), and snapshots were recorded every 50 picoseconds (ps), resulting in a total of 5000 frames. After completing the 100 ns simulations, trajectory files were analyzed to study dynamic conformational changes and interactions within the complexes [76]. Metrics such as root mean square deviation (RMSD), residue root mean square fluctuation (RMSF), Solvent Accessible Surface Area (SASA), and Radius of Gyration (Rg), were computed to assess the stability and behavior of the protein-ligand complexes throughout the simulation. The binding free energy (ΔGbind) of the protein-ligand interactions was calculated for each snapshot using the MM-GBSA method, implemented via the gmx_MM-PBSA tool. To perform the MM-GBSA binding free energy calculation The Desmond trajectory files were converted to GROMACS-compatible formats using Schrödinger utilities, while the corresponding topology files were generated through InterMol conversion of .cms files to .gro and .top formats [77]. The binding free energy (ΔGbind) was calculated using the equation


$$\begin{array}{c} \Delta {\text{G}}_{\text{bind}}=\langle {\text{G}}_{\text{PL}}\rangle -\langle {\text{G}}_{\text{P}}\rangle -\langle {\text{G}}_{\text{L}}\rangle \end{array}$$

In this equation, ⟨GPL⟩, ⟨GP⟩, and ⟨GL⟩ correspond to the average free energies of the complex, the the unbound protein, and the free ligand, respectively

This approach considered various interaction components, such as van der Waals forces, electrostatic interactions, polar solvation effects, SASA contributions, and total binding energies. MD simulation trajectories, performed in explicit water using the Desmond module, were converted to GROMACS-compatible formats via the InterMol tool [78–80]. To further validate the simulation procedure, an additional round of MD simulations was performed using known inactive (negative control) ligands for each target protein under the same simulation protocol. Negative control ligands were retrieved from the ChEMBL [81] database and selected based on their unfavorable docking performance. The resulting protein-ligand complexes were subjected to MD simulations to assess the robustness and reliability of the computational workflow.

### 2.7 Workflow of the entire study

The schematic graphical representation of the entire study given below Fig 2.

**Fig 2:**
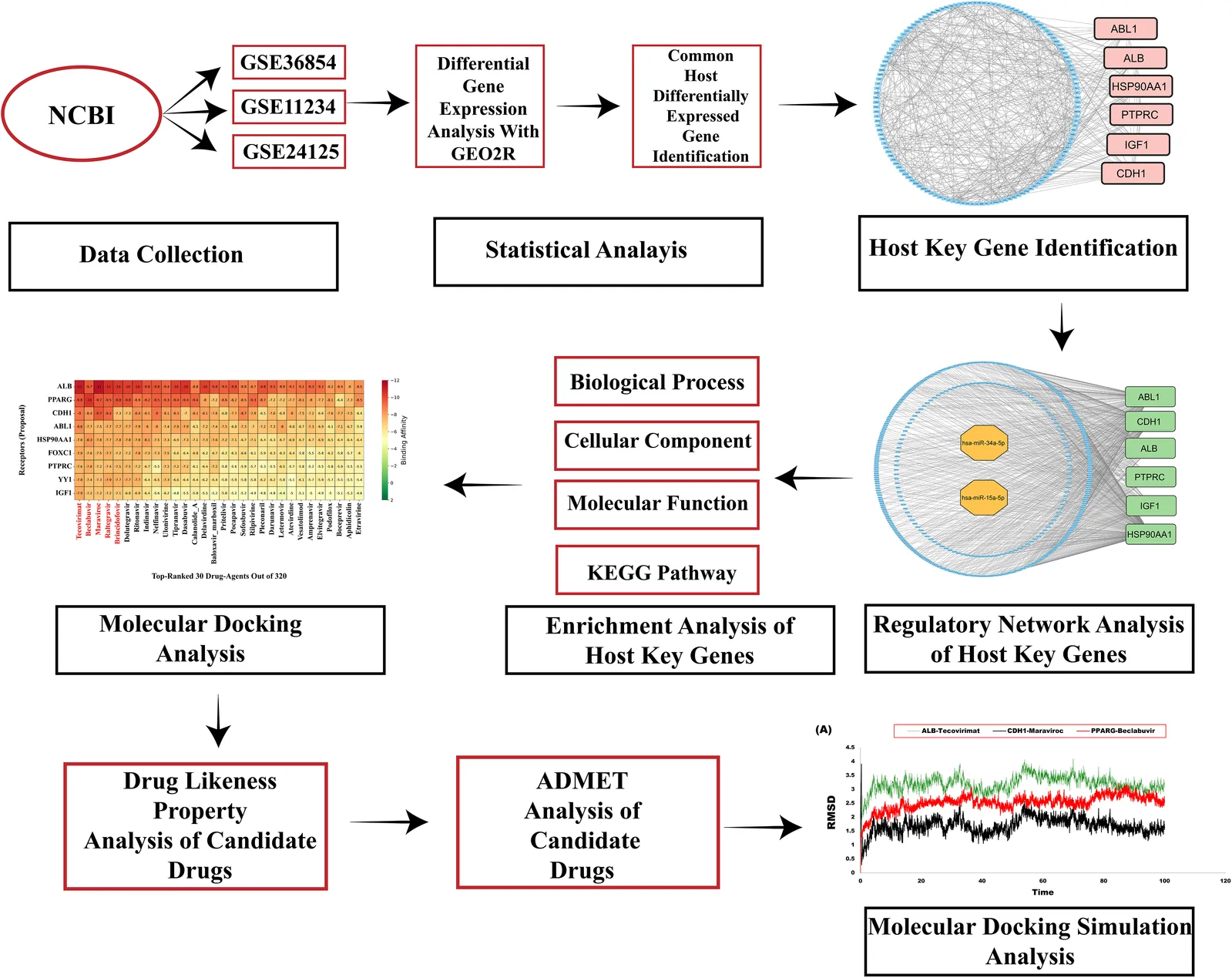
Schematic graphical workflow of the entire study.

## 3 Results

### 3.1 Identification of host DEGs (hDEGs) from transcriptomics profiles

In order to disclose pathogenic processes of MPOX, at first, we aimed to identify overall stable common host differentially expressed genes (hDEGs) between MPOX and control groups, that are independent of age, sex and other environmental variations. We identified DEGs from each of three datasets (GSE36854, GSE24125 and GSE11234) separately to avoid batch and heterogeneity effects that are arisen in the merge data from different environmental sources as provided subsequently. Host DEGs (hDEGs) between MPOX and control samples were identified from each of the three datasets using the statistical LIMMA method. The selection criteria for upregulated hDEGs were an adjusted *p*-value (adj. *p*.val) of 0.05 and log fold change (logFC) > 1, while downregulated hDEGs were defined by an adj. *p*.val of 0.05 and logFC < -1. From datasets GSE11234, GSE36854, and GSE24125, we selected 1617, 1255, and 1523 upregulated DEGs, and 7097, 3345, and 6432 downregulated DEGs respectively. Following this, 75 upregulated and 91 downregulated hDEGs were commonly observed across three datasets, yielding a total of 166 common hDEGs (chDEGs) (S3 Table).

### 3.2 MPOX-causing Host Key Genes (hKGs) Identification from chDEGs

A protein-protein interaction (PPI) network was constructed using the identified chDEGs, resulting in a network comprising 166 nodes and 3,370 edges. To identify host key genes (hKGs) associated with MPOX in a robust and unbiased manner, multiple topological centrality measures implemented in the CytoHubba plugin were applied independently, including maximal clique centrality (MCC), degree, closeness, radiality, maximum neighborhood component (MNC), and edge percolated component (EPC). Each of these metrics captures distinct aspects of node importance within the network. There is no fix threshold of centrality scores for selecting important genes. However, larger centrality scores with each measure is used to select the important genes [82,83]. Therefore, average ranking of host-genes across six topological centrality measures (Degree, Closeness, EPC, MNC, Radiality, and MCC) were used to find top-ranked chDEGs (ALB, HSP90AA1, CDH1, PTPRC, IGF1, IGF1R, ICAM1, and ABL1) (S4 Table). To assess the stability of hKGs, we performed bootstrap resampling of the PPI network over 100 replications using the Python NetworkX library that ordered the genes by three centrality measures (degree, closeness, and betweenness) independently. Then we observed that six genes (ALB, HSP90AA1, CDH1, PTPRC, IGF1, and ABL1) were appeared as the members of top eight genes in at least 80 out of 100 replications based on at least two centrality measures, which were considered as the stable hKGs for further investigations (Fig 3 and S5 Table).

**Fig 3:**
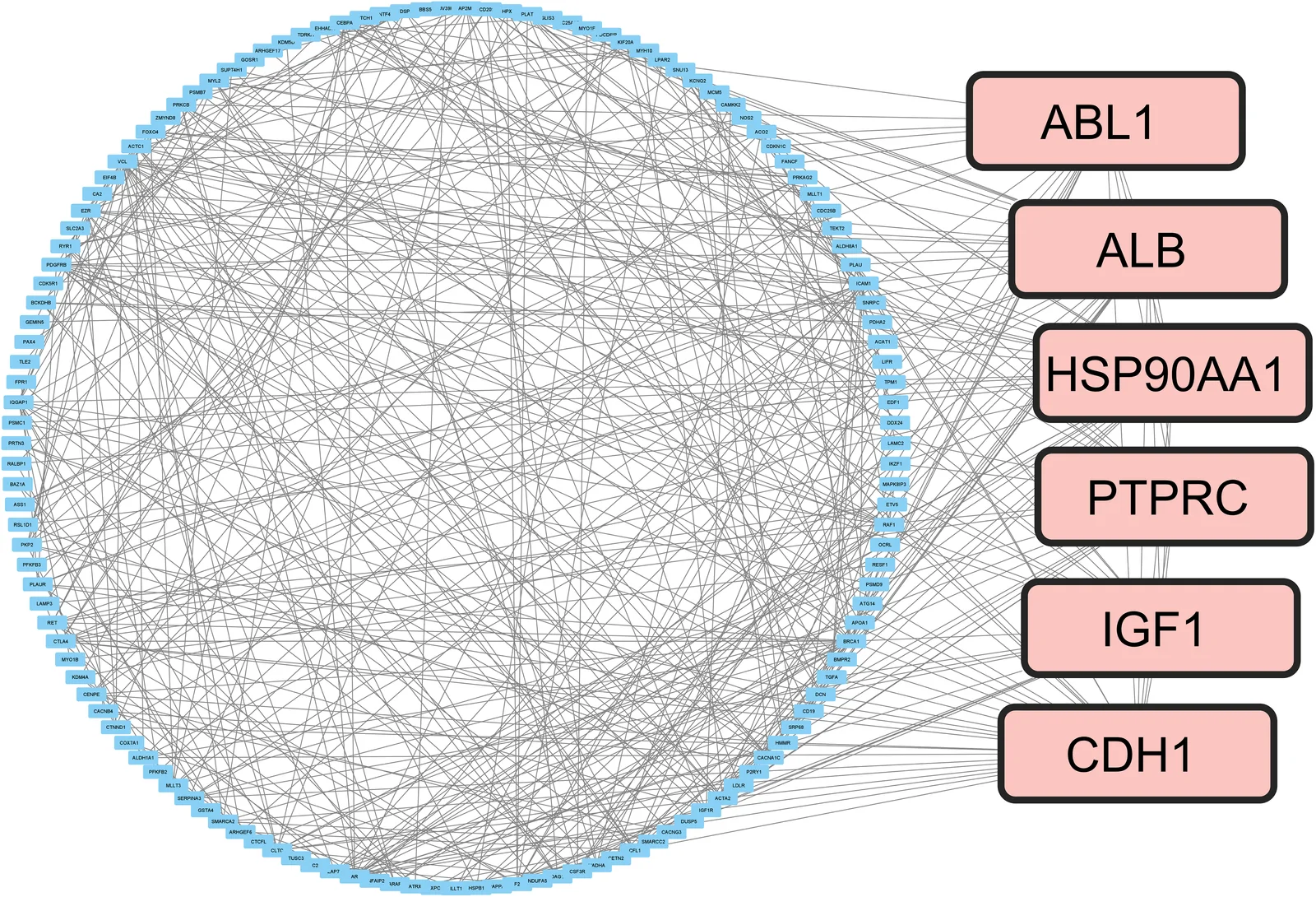
Protein-protein interaction network of common differentially expressed genes highlighting the identified hKGs as pink-rose rectangular.

### 3.3 Identification of key regulators for hKGs

To detect transcriptional and post-transcriptional regulators of the hKGs, we investigated the interaction of hKGs with the transcription factors (TFs) and miRNAs. Through hKGs-TFs interaction network analysis, top-ranked three TFs proteins: FOXC1, PPARG and YY1, were selected as the transcriptional regulators of hKGs by using the average rank of degree and betweenness centrality scores (see Fig 4 and S6 Table), since these three TFs combinedly regulates all genes of hKGs. Similarly, top-ranked two miRNAs: hsa-mir-15a-5p and hsa-mir-34a-5p, were selected as the post-transcriptional regulators of the hKGs (see Fig 5 and S7 Table).

**Fig 4:**
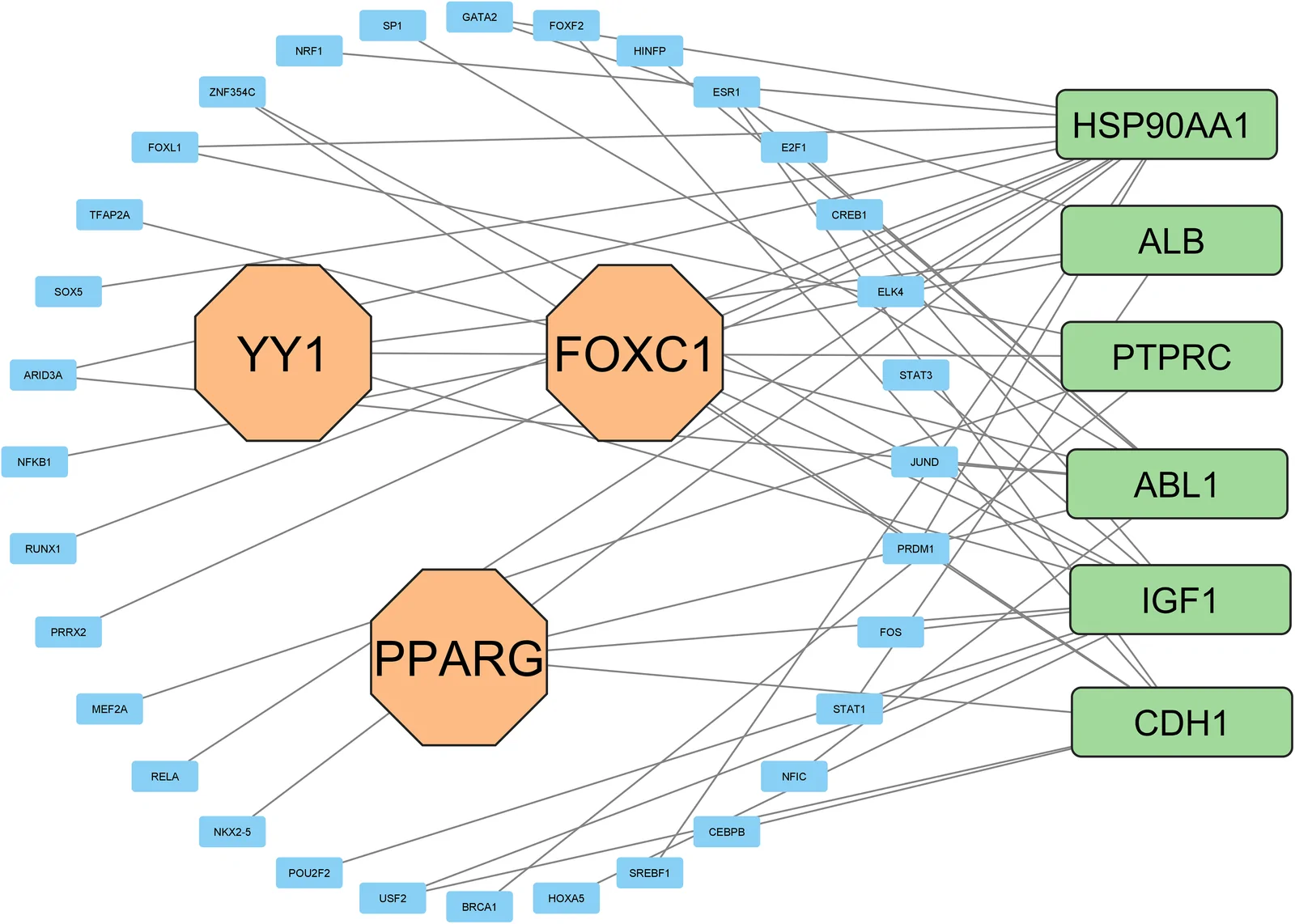
TFs-hKGs interaction network. The three most significant TFs are highlighted in orange-tan, hKGs are shown in green, and other interacting TFs are displayed in blue. Edges represent regulatory interactions between TFs and hKGs.

**Fig 5:**
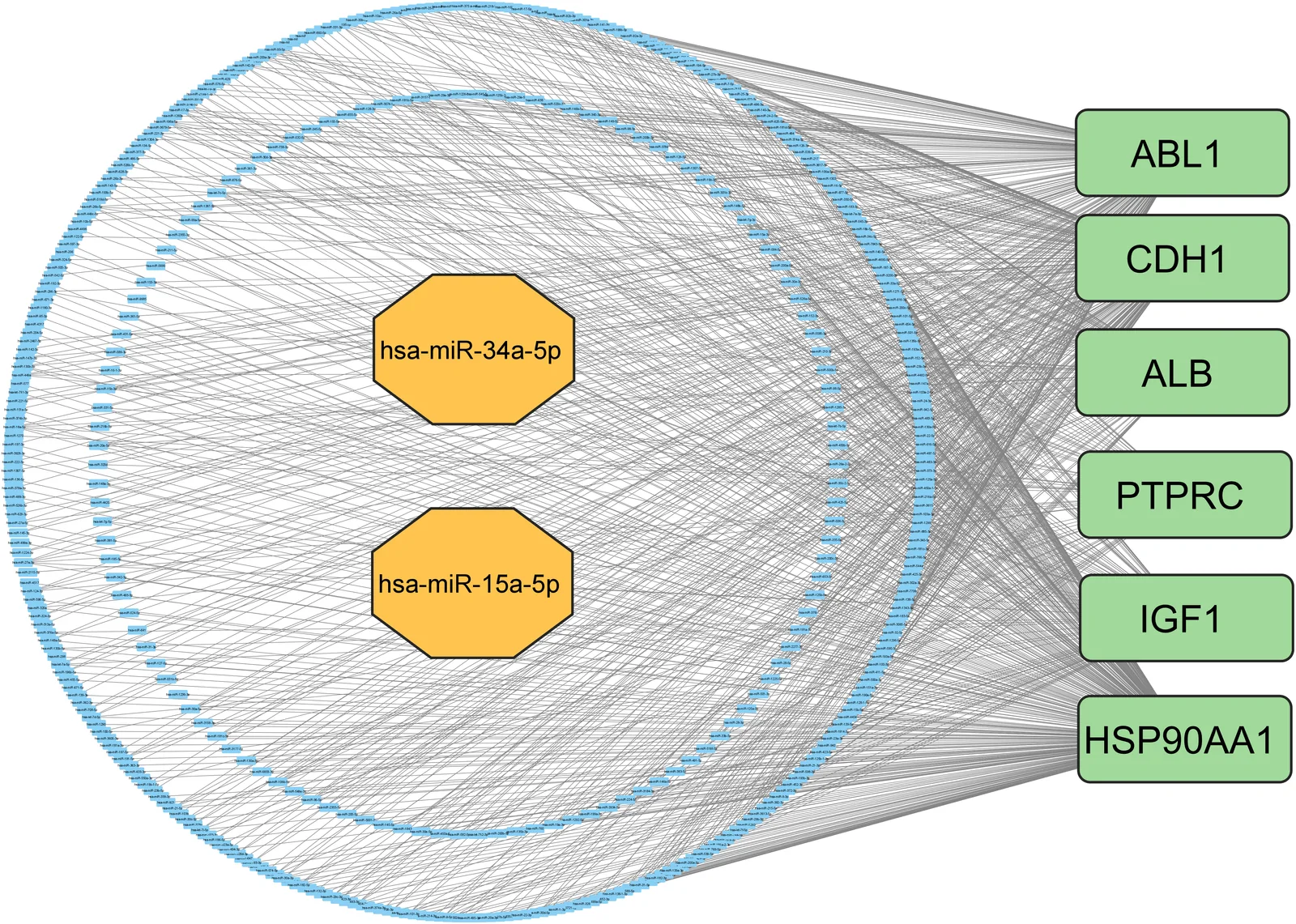
miRNAs-hKGs interaction network. The two most significant miRNAs are highlighted in golden-brown; hKGs are shown in green, and other interacting microRNAs are displayed in blue. Edges represent regulatory interactions between miRNAs and hKGs.

### 3.4 GO and KEGG Enrichment Analysis of hKGs

GO and KEGG enrichment analyses were performed to uncover the key biological roles of hKGs relevant to MPOX infection.

The analysis revealed that the hKGs are primarily involved in regulating key cellular processes such as MAPK and ERK1/ERK2 signaling cascades, which are critical for the inflammatory and immune responses. The hKGs are also enriched in cellular components like the extracellular exosome and plasma membrane, which play a vital role in viral entry and cell-to-cell communication. Additionally, molecular functions such as receptor binding and kinase activity are highlighted, indicating their role in signaling and immune response modulation. KEGG pathway analysis also highlighted key roles in pathways such as cell adhesion molecules, PI3K-Akt signaling, and Ras signaling, emphasizing their significance in host-pathogen interactions and cellular defense responses against MPOX (Table 1).

**Table 1:** Top five enriched GO terms and KEGG pathways for hKGs based on statistical significance (*p-*value < 0.05).

Biological Processes		
GO term	*p*-value	Annotated sKGs
GO:0070374 ~ positive regulation of ERK1 and ERK2 cascade	5.15E-05	PTPRC, ABL1, IGF1
GO:0050731 ~ positive regulation of peptidyl-tyrosine phosphorylation	3.71E-04	PTPRC, ABL1, IGF1
GO:0043410 ~ positive regulation of MAPK cascade	0.00183714	PTPRC, IGF1
GO:0098609 ~ cell-cell adhesion	0.00239335	CDH1, ABL1
GO:0010468 ~ regulation of gene expression	0.00280332	PTPRC, CDH1, IGF1
**Cellular Component**		
**GO term**	***p*-value**	**Associated KGs**
GO:0035867 ~ alphav-beta3 integrin-IGF-1-IGF1R complex	0.00133996	IGF1
GO:0070062 ~ extracellular exosome	0.00364129	HSP90AA1, PTPRC, CDH1, ALB
GO:0005886 ~ plasma membrane	0.01745858	HSP90AA1, PTPRC, CDH1, ABL1
GO:0009986 ~ cell surface	0.01867753	HSP90AA1, PTPRC
GO:0016020 ~ membrane	0.06310227	HSP90AA1, PTPRC, CDH1
**Molecular Function**		
**GO term**	***p*-value**	**Associated KGs**
GO:0030506 ~ ankyrin binding	0.00687051	PTPRC, CDH1
GO:0005158 ~ insulin receptor binding	0.00795162	IGF1
GO:1990782 ~ protein tyrosine kinase binding	0.02191454	HSP90AA1, CDH1
GO:0004713 ~ protein tyrosine kinase activity	0.03711398	ABL1
GO:0005178 ~ integrin binding	0.05729454	IGF1
**KEGG Pathway**		
**KEGG function**	***p*-value**	**Associated KGs**
hsa04514: Cell adhesion molecules	0.00620757	PTPRC, CDH1
hsa04015: Rap1 signaling pathway	0.01110310	CDH1, IGF1
hsa04014: Ras signaling pathway	0.01386299	ABL1, IGF1
hsa04151: PI3K-Akt signaling pathway	0.03063459	HSP90AA1, IGF1
hsa04520: Adherens junction	0.07138208	PTPRC, CDH1

#### 3.4.1 Exploring candidate drugs by molecular docking.

Eight hKGs and their regulating TFs were considered as potential drug target receptors for MPOX. The 3D structures of the 7 receptors (ALB, YY1, ABL1, PPARG, IGF1, PTPRC, and HSP90AA1) were obtained from the Protein Data Bank (PDB) with their respective PDB codes: 6FAK, 1ZNM, 1AB2, 1ZGY, 1B9G, 5FN7, and 8B7J. two receptors (FOXC1, and CDH1) were sourced from the AlphaFold Protein Structure Database using their UniProt IDs: Q12948, and Q9H665, respectively. Molecular docking analysis was performed between the selected receptors and a set of meta-drug agents (S8 Table) to determine their binding affinity scores (BAS). To identify potential candidate drugs, the receptors were ranked in descending order based on the row sums of the binding affinity matrix B=(S*ij*), while the drug agents were ranked according to the column sums. The analysis revealed that the top five drugs (Tecovirimate, Beclabuvir, Maraviroc, Raltegravir, and Brincidofovir) exhibited significant binding affinities score greater than the standard BAS (-7.0 kcal/mol) across all receptors (Fig 6). These five lead compounds are therefore considered promising candidates for the treatment of MPOX. Additionally, we exhibited the drug-target binding positions using AutoDock Vina, with 2D schematic diagrams of receptor-ligand interactions and 3D views of the complexes provided in Table 2, highlighting interacting residues.

**Table 2:** Three highest-ranked drug–target complexes with their corresponding 3-dimensional representations and key interacting residues.

Protein & Ligand complex	Binding Affinity (kCal/ mol)	The 3D View of Complexes	Target-Ligand Interaction highlighting targeted residues
ALB & Tecovirimat	-11.1	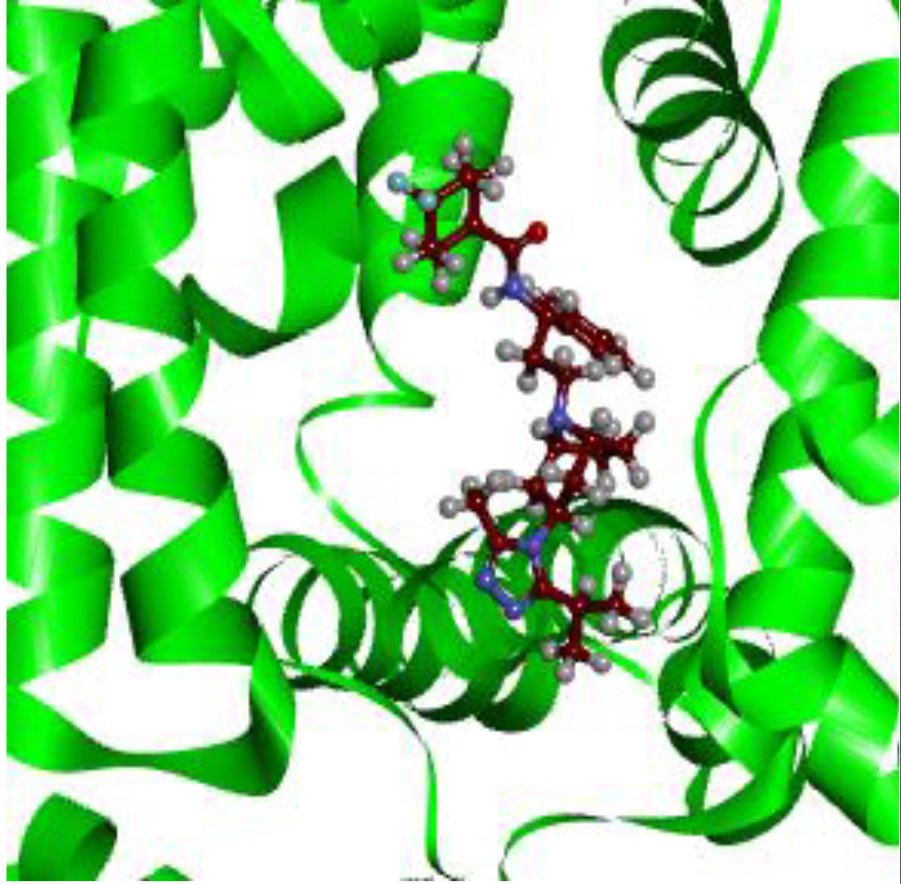	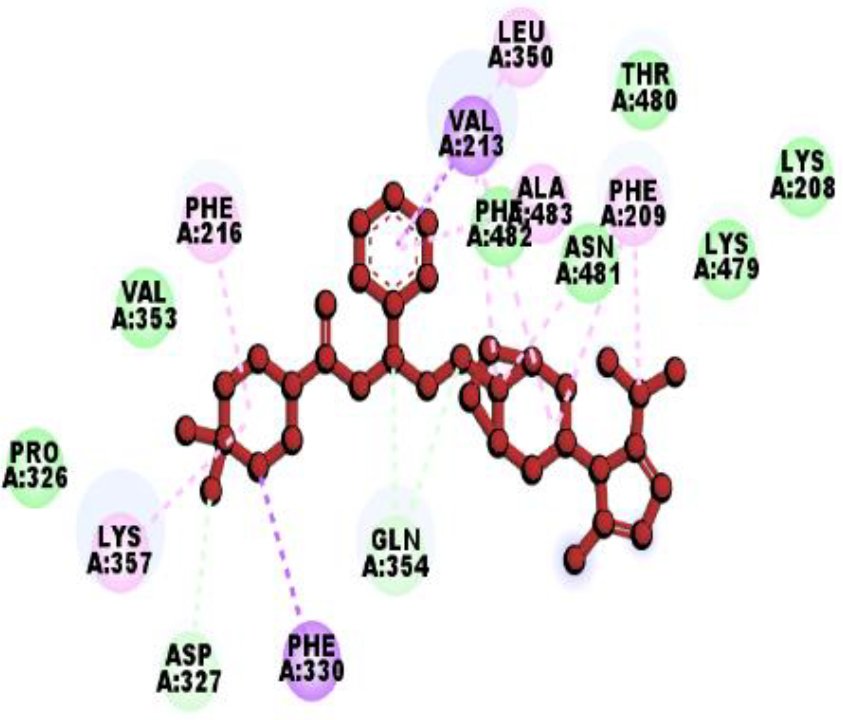
PPARG & Beclabuvir	-10.5	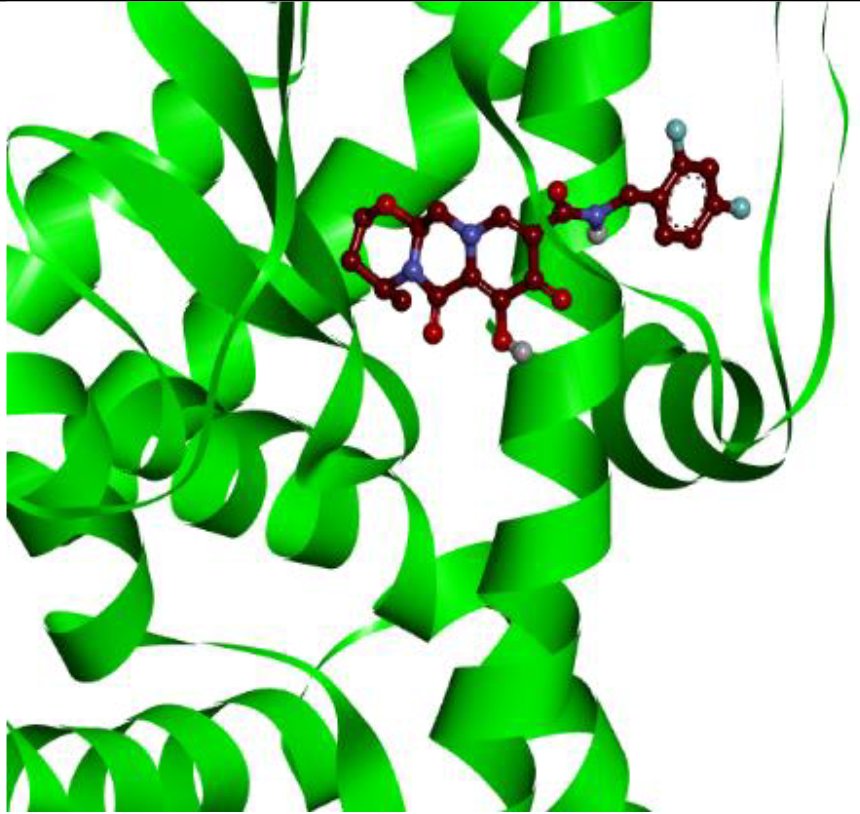	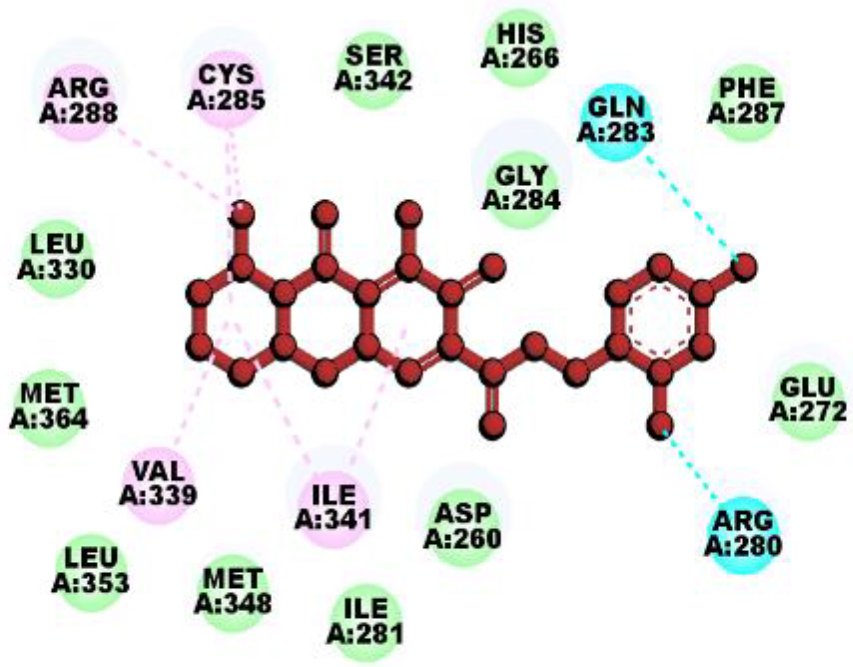
CDH1 & Maraviroc	-9.7	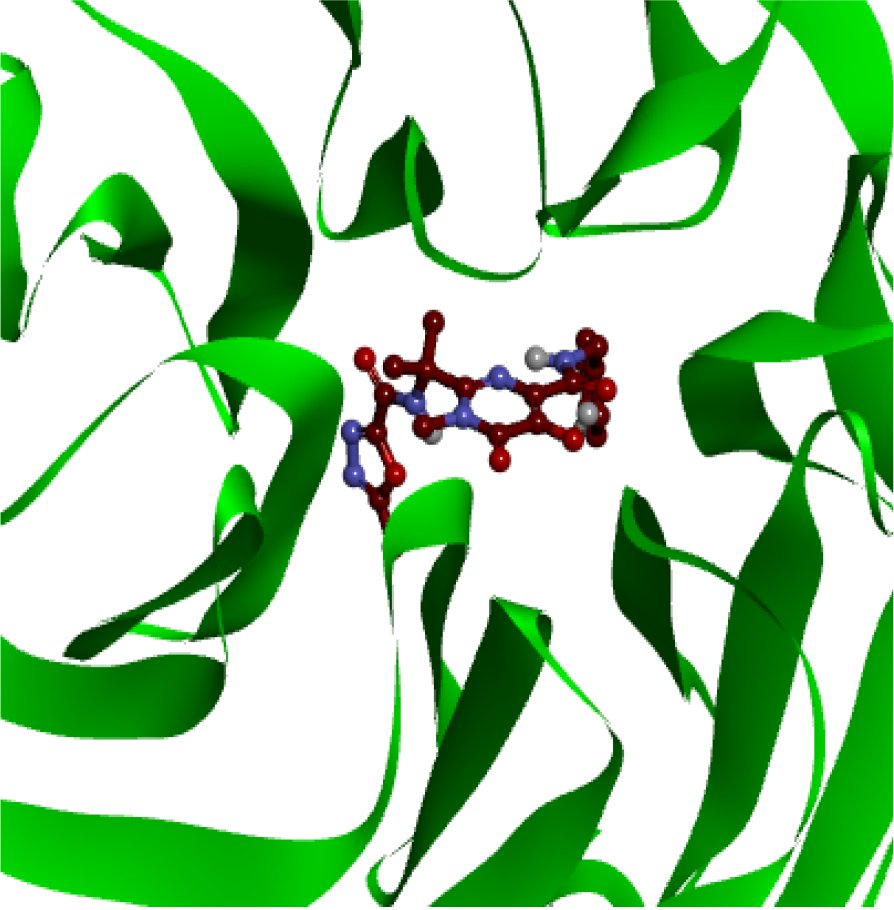	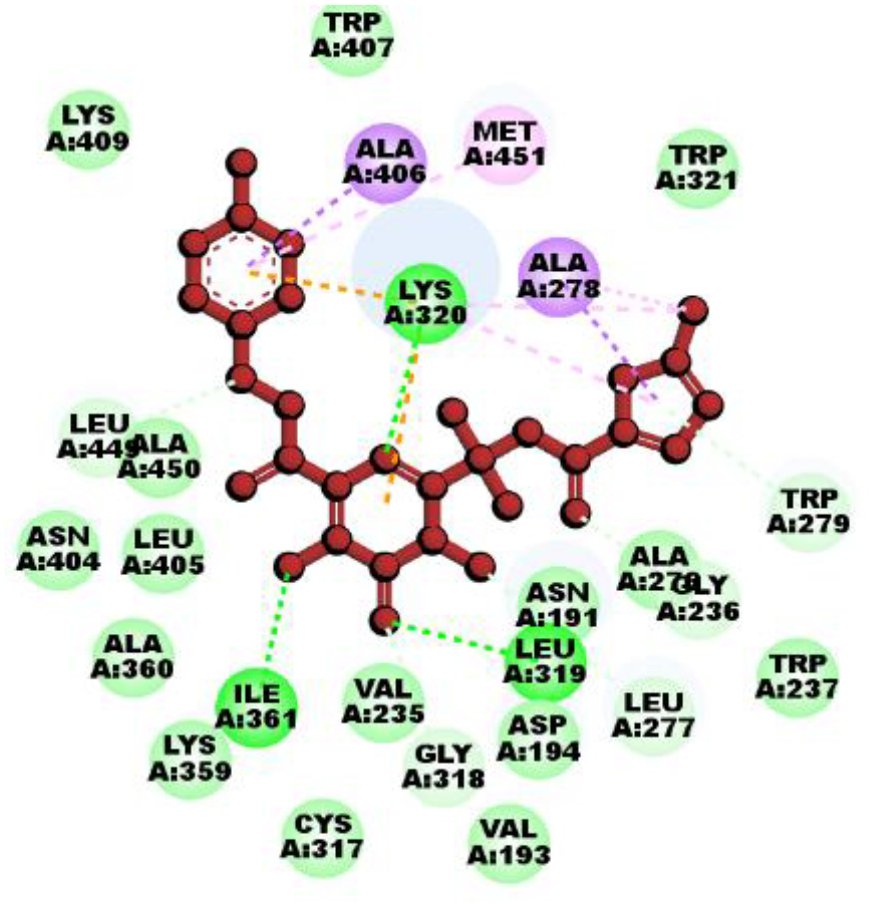

**Fig 6:**
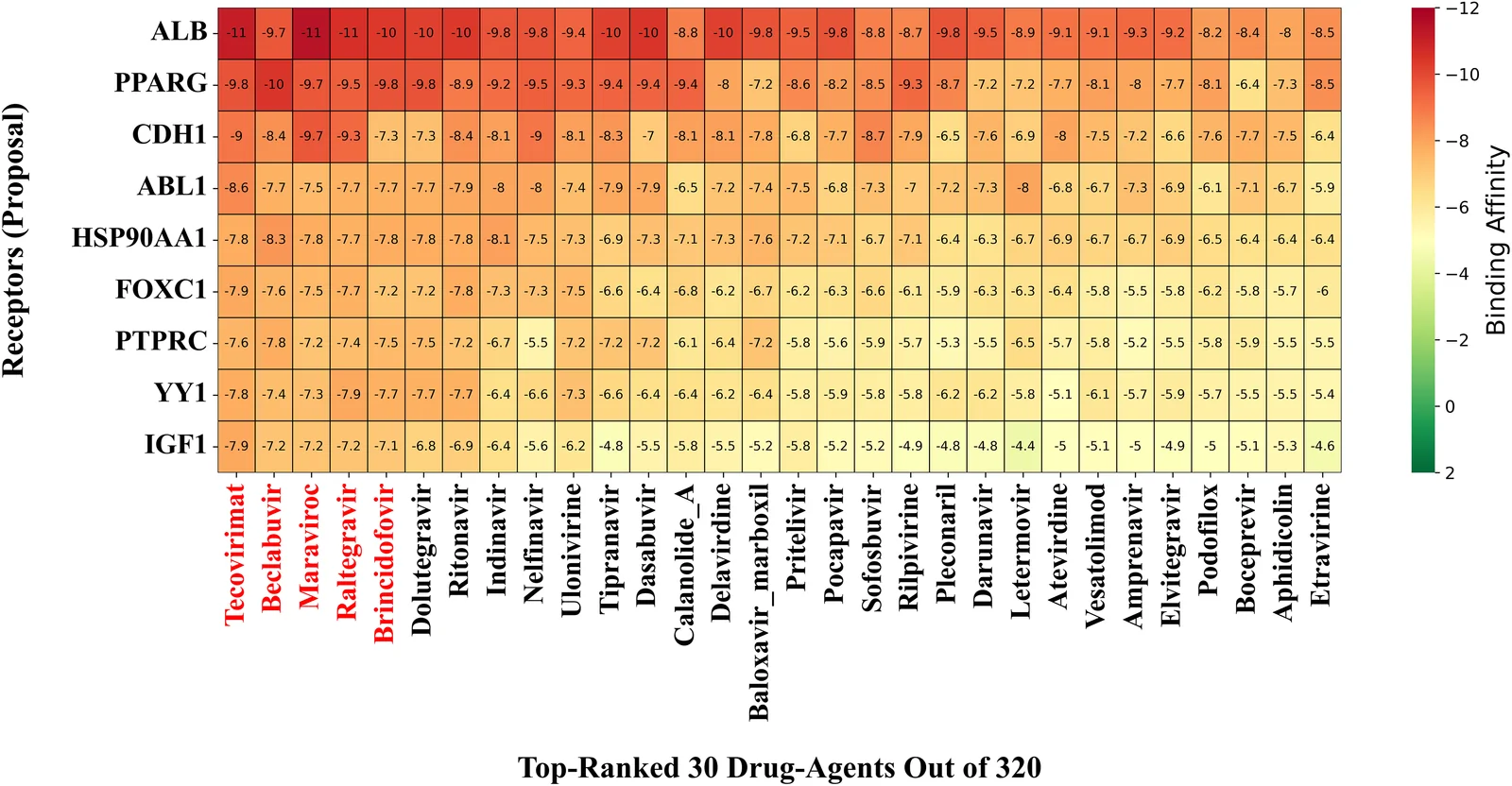
Binding affinity heatmap of the top 30 repurposed drug candidates against the identified receptor proteins.

#### 3.4.2 Check Drug-likeness Properties.

The top five candidate drugs (Tecovirimat, Beclabuvir, Maraviroc, Raltegravir, and Brincidofovir) were evaluated for drug-likeness based on Lipinski’s Rule of Five (RO5). This rule assesses key parameters, including molecular weight, lipophilicity (LogP), hydrogen bond donors (HBD), and hydrogen bond acceptors (HBA), to determine the drug-like nature of a compound. Drug-like molecules typically require a logP ≤ 5 and a molecular weight ≤ 500 g/mol to ensure good membrane permeability and absorption. Although some compounds had minor RO5 violations, all five candidates exhibited suitable pharmacokinetic properties and bioavailability. These findings suggest that the selected molecules meet most of the essential criteria for drug-likeness and possess favorable pharmacokinetic characteristics, making them promising candidates for further optimization and drug development (Table 3).

**Table 3:** Drug likeness properties of top ranked 5 candidate drug molecules.

Compounds	Molecular weight (MW)	Log P	HBA	HBD	Rotatable bond (ROTB)	Bioavailability Score	Lipinski’s Rule follow
	SB ≤ 500	SB ≤ 5	SB ≤ 10	SB ≤ 5	SB ≤ 5		
Tecovirimat	376.33	2.61	6	1	4	0.55	5
Beclabuvir	659.84	4.54	7	1	4	0.55	4
Maraviroc	513.67	4.15	6	1	3	0.55	4
Raltegravir	444.423	0.91	9	3	6	0.55	4
Brincidofovir	561.69	5.00	9	3	26	0.55	3

N.B: HBA = Hydrogen bond acceptor, HBD = Hydrogen bond donor, SB = Should Be

#### 3.4.3 Pharmacokinetics (PK) and Toxicity analysis.

The pharmacokinetic and toxicity profiles of the candidate compounds were evaluated to assess their suitability as potential therapeutic agents. Key ADME/T parameters, including absorption, distribution, metabolism, excretion, and toxicity, were analyzed alongside drug-likeness properties (Table 4). Oral bioavailability is strongly influenced by intestinal absorption. Human Intestinal Absorption (HIA) values greater than 30% are generally considered indicative of efficient uptake. In this study, all compounds exhibited high HIA values (≥68%), suggesting favorable absorption characteristics. Similarly, Caco-2 permeability, an indicator of intestinal transport, showed acceptable values for all candidates (greater than -5.15 log cm/s), supporting their potential for oral administration [64,84,85]. The ability of compounds to penetrate the blood-brain barrier (BBB) was assessed using logBB values. Compounds with logBB values below -1 are considered poorly permeable, whereas values above 0.3 indicate effective penetration [86]. None of the selected compounds demonstrated strong BBB permeability, as all values remained below 0.3, suggesting limited central nervous system exposure [29,30]. Drug distribution was evaluated using the volume of distribution at steady state (VDss). The observed VDss values indicated adequate distribution in systemic circulation. In addition, central nervous system permeability, estimated using LogPS values, suggested partial penetration into the CNS [84], and our compounds showed excellent VDss, meaning good plasma distribution. Drug metabolism was examined through interactions with cytochrome P450 (CYP) enzymes, which play a key role in biotransformation. Toxicological assessment, including AMES testing and skin sensitization analysis, indicated that the selected compounds are non-mutagenic and unlikely to cause skin-related adverse effects [87,88]. Overall, these results suggest that the candidate compounds possess favorable pharmacokinetic profiles with acceptable safety characteristics, supporting their potential for further investigation.

**Table 4:** ADMET analysis of selected top-ranked candidate drug molecules using different web tools.

Compounds	Absorption			Distribution		Excretion				Toxicity				
	LogS	HIA (%)	Caco2	BBB	VDss	CYP1A2 Inhibitor	CYP3A4 inhibitor	CYP2C9 Substrate	Half Life (T_1/2_)	Total Clearance	AMES Toxicity	Rat Oral Acute Toxicity	Skin Sensitization	Carcinogenicity
Tecovirimat	-4.65	97.2	1.00	-0.05	0.07	No	No	0.81	1.83	0.80	No	2.80	No	0.44
Beclabuvir	-4.65	97.4	1.33	-0.84	0.04	No	No	0.83	6.99	0.71	No	2.60	No	0.09
Maraviroc	-5.91	91.3	0.95	0.04	0.83	No	No	0.43	4.01	0.72	No	3.15	No	0.18
Raltegravir	-4.16	68.1	-0.36	-1.62	-1.6	No	No	0.56	6.05	0.62	No	2.28	No	0.17
Brincidofovir	-4.80	68.4	-0.37	-2.34	-0.3	No	Yes	0.75	2.75	0.91	No	2.64	No	0.42

#### 3.4.4 Connectivity Map analysis of prioritized drug candidates.

To provide computational assessment of the prioritized compounds, CMap analysis was performed using the chDEGs. Among the five prioritized compounds, Maraviroc and Raltegravir exhibited negative normalized connectivity scores (NCS <−1) across multiple human cell lines, including A549, HeLa, and HEK293 (S9-S10 Tables). Negative connectivity scores indicate that the transcriptional signatures induced by these compounds are inversely associated with the monkeypox-associated gene expression signature. In contrast, Tecovirimat, Beclabuvir, and Brincidofovir did not show comparable connectivity under the applied selection criteria.

#### 3.4.5 Molecular Dynamics Simulation.

Molecular dynamics (MD) simulations were employed to examine the structural stability and interaction profiles of three selected protein-ligand complexes: ALB-Tecovirimat, CDH1-Maraviroc, and PPARG-Beclabuvir (see Fig 7). The ALB-Tecovirimat complex demonstrated good structural stability, with an RMSD (root mean square deviation) of 3.09 Å, reflecting moderate fluctuations around its equilibrium state. The RMSF (root mean square fluctuation) of 1.53 Å further supported this stability, while the binding free energy of -38.75 kJ/mol indicated a favorable binding interaction between Tecovirimat and the ALB protein. The CDH1-Maraviroc complex exhibited the highest degree of stability among the three, with an RMSD of 1.78 Å, suggesting minimal deviation from its initial conformation during the simulation. Its RMSF of 2.42 Å showed acceptable residue fluctuations, and the notably high binding affinity, with a binding free energy of -45.57 kJ/mol, suggested a strong and stable interaction between CDH1 and Maraviroc. For the PPARG-Beclabuvir complex, stability was confirmed by an RMSD of 2.51 Å, which indicated relatively consistent behavior throughout the simulation. The RMSF of 1.12 Å highlighted minimal fluctuations in specific residues. The binding free energy of -36.24 kJ/mol further supported that Beclabuvir formed a stable interaction with PPARG. Overall, these results suggest that all three complexes are stable and exhibit favorable binding interactions. In order to validate the simulation procedure, we performed MD simulation of PPARG receptor with its negative control ligand and observed that RMSD of PPARG with the proposed ligand ‘Beclabuvir’ much smaller than the PPARG with its negative control ligand throughout the 100ns, which indicates the simulation procedure is valid (S1 Fig in S1 File).

**Fig 7:**
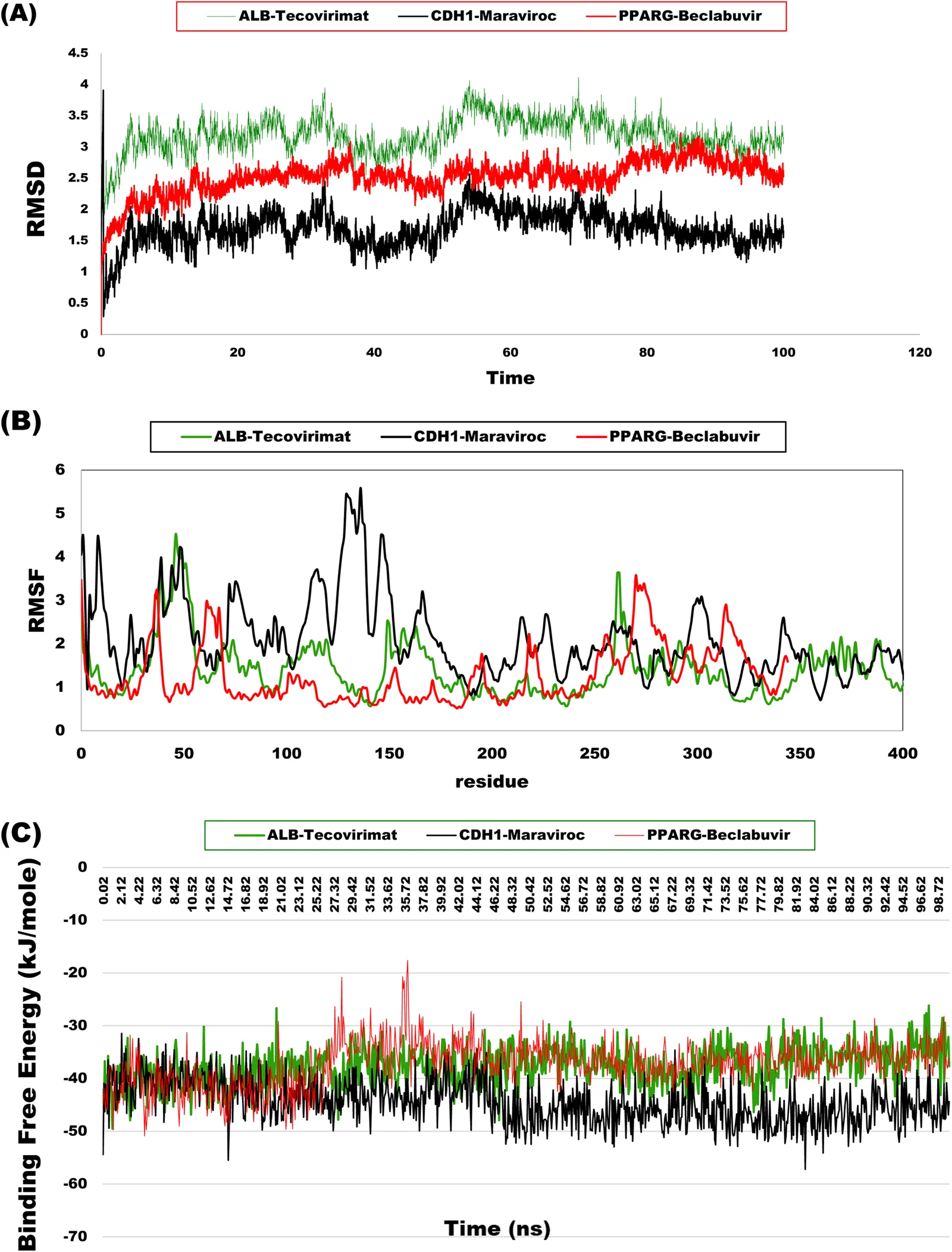
The MD plots where (A) The RMSD of backbone atoms (B) The RMSF of protein-ligand complex (C) Binding free energy of three complexes during MD simulation.

## 4 Discussion

Monkeypox (MPOX) has emerged as a global public health concern due to its increasing incidence and sustained human-to-human transmission, underscoring the need to better understand the host molecular mechanisms underlying disease pathogenesis and progression. Transcriptomic profiling provides a powerful framework for characterizing host responses to viral infections by revealing dysregulated immune pathways, signaling networks, and potential therapeutic targets. In this study, an integrative systems biology approach was applied to investigate host transcriptomic alterations associated with MPOX. Rather than focusing on individual genes alone, our analysis highlights coordinated changes in immune regulation, cell survival, and virus-host interaction pathways that are central to MPOX pathogenesis.

The biological relevance of the identified host key genes (hKGs) to MPOX pathogenesis can be understood through their roles in Orthopoxvirus entry, immune modulation, and host cell survival. HSP90AA1 is a molecular chaperone essential for the stabilization and folding of host and viral proteins. Orthopoxviruses, including MPOX, rely on host chaperone machinery to support viral protein maturation, assembly, and replication [89]. By stabilizing key signaling proteins and suppressing apoptosis through pathways such as PI3K-Akt, HSP90AA1 may help create a cellular environment that favors sustained MPOX replication [90]. Viruses such as Influenza and HIV exploit HSP90 to stabilize and fold their viral proteins, enhancing their replication and survival [89]. Its role in the PI3K-Akt signaling pathway suggests its involvement in cell survival mechanisms that viruses manipulate to prevent apoptosis, thereby allowing prolonged viral persistence in host cells [91]. Its regulation by FOXC1 highlights its strategic importance during cellular stress responses in viral infection [92]. *IGF1* are key regulators of cell survival and immune response modulation [93]. During viral infections, IGF1 signaling may be hijacked by viruses to evade host immune defenses and sustain infected cell viability [93]. The Ras and PI3K-Akt signaling pathways, where these genes are involved, are known to be manipulated by viruses such as hepatitis and Epstein-Barr virus to promote cellular survival and viral replication [94]. The regulation of these genes by FOXC1, PPARG, and YY1 points to their critical role in viral pathophysiology, where they help infected cells resist apoptosis and allow for sustained viral propagation [95]. Collectively, these gene-specific functions align with known MPOX mechanisms involving immune evasion, sustained host cell viability, and enhanced viral spread, supporting the biological relevance of the identified hKGs in MPOX pathogenesis.It’s regulation by FOXC1 and YY1 transcription factors likely fine-tunes immune cell trafficking and adhesion during viral invasion, helping the immune system respond effectively to viral threats [96]. PTPRC is essential for activating immune responses, particularly in T cells, which are critical for clearing viral infections [97]. Alterations in its expression or function can lead to weakened immune responses, allowing viruses to persist. Altered PTPRC activity during MPOX infection may impair lymphocyte activation and immune clearance, contributing to viral persistence and the characteristic immune dysregulation observed in MPOX patients. Its participation in the ERK1/ERK2 cascade and peptidyl-tyrosine phosphorylation processes underscores its role in T-cell signaling during viral infections, where it helps initiate immune reactions against infected cells [98]. Regulation by YY1 positions PTPRC as a key modulator of immune cell activation in response to viral infections [99]. ABL1 is a tyrosine kinase that responds to cellular stress, including viral infections. Some viruses, such as HIV and Epstein-Barr virus, exploit ABL1 to enhance their replication and prevent the host’s antiviral responses [100]. Dysregulated ABL1 signaling may therefore facilitate MPOX intracellular movement and promote viral spread within infected tissues. Its role in the Ras signaling pathway suggests that it aids in the regulation of immune responses, inflammation, and cell survival during viral infections [101]. Its regulation by PPARG links it to pathways involved in cell survival and immune modulation, crucial for both viral replication and host defense. CDH1, a critical component in maintaining epithelial integrity, is often disrupted during viral infections to facilitate viral dissemination. Viruses like HPV and adenovirus disrupt CDH1-mediated cell-cell adhesion to promote their spread across tissues [102]. In viral infections, CDH1’s involvement in adherens junctions and cell adhesion molecules pathways is essential for maintaining tissue barriers and preventing excessive viral entry [102]. In MPOX infection, which involves skin and mucosal lesions, altered CDH1 expression may contribute to compromised tissue integrity, enhanced viral dissemination, and increased susceptibility of neighboring cells to infection. Its regulation by FOXC1 and PPARG during infection underscores its role in preserving tissue structure while potentially limiting viral invasion [103]. ALB is a plasma protein that plays roles in nutrient transport, detoxification, and maintaining osmotic balance. ALB levels are frequently abnormal in viral infections like hepatitis because of inflammatory processes or liver damage [104]. ALB’s association with extracellular exosomes suggests its involvement in intercellular communication during viral infections, where exosomes may carry viral antigens or mediate immune signaling [104]. During MPOX infection, systemic inflammation and liver involvement have been reported, and altered ALB levels may reflect disease severity and inflammatory burden. In addition, ALB-associated extracellular vesicles may contribute to immune modulation and intercellular signaling during MPOX infection, potentially facilitating viral dissemination or host response dysregulation [105]**.** Regulation by YY1 highlights its role in viral infection-associated inflammation and immune modulation [104]. Importantly, many of the mechanisms associated with these hKGs align with known aspects of MPOX pathogenic. Orthopoxviruses, including the MPOX virus, rely on host chaperones, survival signaling pathways, and adhesion molecules to facilitate viral entry, replication, and immune evasion. For example, MPOX virus entry involves host membrane fusion and endocytic processes, which are closely linked to cell adhesion molecules and cytoskeletal regulation, implicating gene CDH1 [106,107]. In addition, MPOX is known to modulate host PI3K-Akt, MAPK, and ERK signaling pathways to suppress apoptosis and prolong host cell survival, consistent with the involvement of HSP90AA1, IGF1, and ABL1 identified in this study [108,109]. Dysregulation of immune signaling and lymphocyte activation is a hallmark of MPOX infection, particularly during systemic disease, supporting the relevance of immune-associated genes such as PTPRC [110]. Collectively, these findings suggest that the identified host key genes are not only central in viral infections broadly but are also mechanistically consistent with established features of MPOX viral entry, immune evasion, and host response. Additionally, the overall GO/KEGG enrichment analysis suggests that the identified hKGs reflect host programs plausibly engaged in MPOX rather than generic antiviral responses.Enrichment of MAPK/ERK, PI3K-Akt, and upstream tyrosine-kinase signaling (ABL1, IGF1, PTPRC, HSP90AA1) aligns with orthopoxvirus models demonstrating kinase activation as a requirement for efficient viral replication.

Binding affinity screening between 320 candidate compounds and hKG-associated targets identified five promising repurposable drugs: Tecovirimat, Beclabuvir, Maraviroc, Raltegravir, and Brincidofovir. These candidates demonstrated favorable drug-likeness and ADMET profiles, while molecular dynamics simulations of the top three complexes confirmed stable protein-ligand interactions. Among these, Beclabuvir is a non-nucleoside inhibitor of the NS5B RNA polymerase, a critical enzyme in hepatitis C virus (HCV) replication [111]. By blocking the viral RNA polymerase, it prevents the replication of HCV, effectively lowering viral levels in patients [112]. Beclabuvir’s mechanism of inhibiting RNA polymerase may be valuable in targeting the replication machinery of the MPOX, which relies on its own DNA polymerase. Although MPOX is a DNA virus and HCV is an RNA virus, targeting viral enzymes involved in genome replication could provide therapeutic benefits. Maraviroc is commonly used in combination therapies to manage HIV in patients with CCR5-tropic strains of the virus. Maraviroc’s inhibition of the CCR5 receptor, which plays a role in immune cell infection by HIV, may offer protection against MPOX by modulating immune responses. MPOX infects human cells and may utilize host receptors for entry into these cells, though its exact entry mechanisms differ from HIV. By blocking certain immune cell receptors, maraviroc could reduce the extent of viral entry or alter the immune response in ways that limit the severity of MPOX infection. Additionally, CCR5 has been implicated in inflammation and immune regulation, potentially providing therapeutic benefits by reducing the inflammatory response associated with MPOX infection [113–115]. Raltegravir is also an integrase inhibitor used to treat HIV infection. It inhibits the HIV integrase enzyme, which is necessary for integrating the viral genome into the host DNA, a critical step in the virus’s life cycle [116,117]. Raltegravir could still play a role in inhibiting other steps of the viral replication cycle, especially through unknown interactions with host or viral proteins involved in MPOX replication. However, host targets are generally less likely to be directly affected by viral evolution compared to viral proteins, as they are conserved within the human genome and are not subject to viral mutational pressure. This makes host-directed targets comparatively stable and reliable candidates for therapeutic intervention. Nevertheless, drug efficacy may still be indirectly influenced by viral evolution through alterations in virus-host interaction dynamics. In this context, it is important to emphasize that the molecular docking and molecular dynamics analyses presented in this study are exploratory in nature and are intended to generate testable hypotheses rather than to establish definitive therapeutic efficacy. Notably, Tecovirimat and Brincidofovir, which emerged among the top-ranked compounds in our analysis, have already been approved or investigated for the treatment of MPOX, providing external biological support for the relevance of our computational framework. However, further CMap analysis provided additional transcriptomic support for two of the five prioritized compounds, Maraviroc and Raltegravir, both of which exhibited negative NCS across multiple human cell lines including A549, HeLa, and HEK293. These human cell lines are widely used for antiviral and transcriptomic perturbation studies, as well as in MPOX, providing biologically relevant contexts for evaluating drug-induced transcriptional responses [118–120]. In contrast, the remaining compounds did not demonstrate comparable inverse connectivity under the applied selection criteria due to limitation of the database. However, this observation does not necessarily exclude their therapeutic potential, as CMap evaluates the similarity between drug-induced and disease-associated transcriptional signatures rather than direct antiviral activity or target binding. Furthermore, the LINCS database is generated using specific human cell lines, treatment durations, and drug concentrations, which may not fully capture the biological context of monkeypox infection or the mechanisms of action of all antiviral agents.

The study has several notable strengths. An integrative systems biology framework was employed by combining transcriptomic analysis, protein-protein interaction network construction, regulatory network inference, functional enrichment analysis, drug repurposing strategies, molecular docking, and molecular dynamics simulations. The use of multiple independent datasets and a cross-study consistency approach enhanced the robustness and reproducibility of the identified host key genes. Importantly, several of the prioritized drug candidates have already been approved or clinically investigated for MPOX or other viral infections, providing external biological support for the relevance and translational potential of the proposed computational framework. Furthermore, prioritizing host-directed therapeutic targets offers a complementary strategy to conventional antiviral development, potentially reducing susceptibility to viral mutation and resistance.

This study has several limitations that should be acknowledged. First, the analyses are based entirely on *in-silico* and systems biology approaches and should therefore be interpreted as hypothesis-generating. The identified hKGs, regulatory networks, and repurposable drug candidates represent computational predictions intended to prioritize biologically plausible mechanisms and therapeutic targets. Therefore, these findings require confirmation in appropriate cellular or animal models, as well as validation in clinical cohorts, to establish their biological significance and therapeutic potential in the context of MPOX.

The findings of this study have potential implications for both the diagnosis and treatment of MPOX. The identified host key genes and regulatory signatures may serve as candidate biomarkers for disease detection, severity assessment, or host response monitoring. From a therapeutic perspective, the prioritized repurposable drugs, particularly those supported by existing clinical or experimental evidence, provide a rational starting point for further validation and optimization. Future work integrating these computational predictions with experimental studies, clinical cohorts, and public health surveillance data will be critical for translating these findings into actionable diagnostic tools and effective treatment strategies for MPOX.

## 5 Conclusion

This study provides valuable insights into the molecular mechanisms of MPOX virus infection and identifies potential therapeutic candidates for its treatment. By analyzing transcriptomic profiles from three datasets, we identified 166 common host differentially expressed genes (chDEGs), of which six top-ranked hKGs (ALB, HSP90AA1, CDH1, PTPRC, IGF1, and ABL1) were highlighted as key contributors to MPOX pathogenesis. The hKGs-set enrichment analysis with biological processes, molecular functions, cellular components, KEGG pathways, and regulatory factors (TFs, proteins, and miRNAs) disclosed pathogenic mechanisms. Furthermore, hKGs-guided top-ranked five repurposable drug candidates (Tecovirimat, Beclabuvir, Maraviroc, Raltegravir, and Brincidofovir) were identified as potential inhibitors of MPOX, and their pharmacokinetic properties were confirmed through ADME/T and drug-likeness assessments. Connectivity Map (CMap) analysis identified maraviroc and raltegravir as candidate drug molecules predicted to reverse MPOX-associated transcriptional signatures in A549 and HEK293 cell-line contexts, thereby supporting their prioritization for further experimental evaluation as potential anti-MPOX virus agents. Molecular dynamics simulations confirmed the stability of drug-hKG complexes, reinforcing their potential for therapeutic use. However, the identified hKGs were not validated using experimental methods due to the unavailability of wet-lab facilities. Experimental validation is crucial to confirm the computational results and ensure their biological relevance. Therefore, these *in-silico* findings may provide a foundation for further experimental validation and offer promising strategies for managing MPOX outbreaks.

## Supporting information

S1 File Written agreement for change in authorship with positions. (DOCX)

S1 Table Detail information of the dataset used in the analyses. (XLSX)

S2 Table Collection of drug agents for Monkeypox, smallpox and hKGs-guided drugs agents. (XLSX)

S3 Table List of upregulated and downregulated chDEGs. (XLSX)

S8 Table Docking/ (binding affinity) scores (kcal/mol) between the proposed target genes/proteins (receptors) and top ordered 30 candidate drugs (out of 320). (XLSX)

S5 Table Bootstrap validation results for hub genes (hKGs) selection. (XLSX)

S1 Fig RMSA comparison of PPARG protein with its control native ligand and proposed ligand Beclabuvir. (TIF)

S4 Table Average ranking of host-genes across six topological centrality measures (Degree, Closeness, EPC, MNC, Radialty, MCC) to find top-ranked host-key-genes(hKGs). (XLSX)

S6 Table Average ranking of host-genes across two topological centrality measures (Degree and Betweenness) to find top-ranked TFs proteins as the transcriptional regulators of hKGs. (XLSX)

S7 Table Average ranking of host-genes across two topological centrality measures (Degree and Betweenness) to find top-ranked miRNAs as the post-transcriptional regulators of hKGs. (XLSX)

S9 Table CMap analysis results for drug raltegravir. (XLSX)

S10 Table CMap analysis results for drug maraviroc. (XLSX)
