# Supplementary material for: Disclosing pathogenic processes of monkeypox (MPOX) and drug repurposing through host-transcriptomics analysis
Source: PLoS Negl Trop Dis. 2026 Oct 16;20(10):e0014797. doi: 10.1371/journal.pntd.0014797 (PMC13674749; doi:10.1371/journal.pntd.0014797)
Supplement: Supplementary file 1 [file pntd.0014797.s001.docx]

**WRITTEN AGREEMENT FOR CHANGE IN AUTHORSHIP with Positions**

**Journal:** PLOS Neglected Tropical Diseases
**Manuscript Title:** “Disclosing pathogenic processes of monkeypox (MPOX) and drug repurposing throughhost-transcriptomics analysis”
**Manuscript ID:** “PNTD-D-25-01313R2”

**Proposed Change in Authorship**

We, the undersigned authors of the above-mentioned manuscript, hereby confirm that we have mutually agreed to the following change in the authorship of the manuscript:

**Current author list:**
Sabkat Mahmud, Alvira Ajadee, Md. Ahad Ali, Reaz Ahmmed, Md. Masud Rana, Tasfia Noor, Md. Fahim Faysal, Md. Hadiul Kabir, Md. Nurul Haque Mollah*

**Proposed author list:**
Sabkat Mahmud, Alvira Ajadee, Md. Ahad Ali, Md. Feroj Ahmed, Reaz Ahmmed, Md. Masud Rana. Tasfia Noor, Md. Fahim Faysal, Md. Hadiul Kabir, Md. Nurul Haque Mollah*

**Author(s) being added:**
Md. Feroj Ahmed

**Author(s) being removed:**
None

**Author(s) whose order is being changed:**
Reaz Ahmmed, Md. Masud Rana. Tasfia Noor, Md. Fahim Faysal, Md. Hadiul Kabir, Md. Nurul Haque Mollah*

**Reason for the Authorship Change**

The proposed change is being made because:

This author contributed to the formal analysis, methodology, validation, and visualization during the major revision of the manuscript. These contributions were made during the 2nd major revision of the manuscript; therefore, the author was not included in the original submission. Actually, the first two authors are now absent at our university, they are now doing PhD research at Tulane University, USA. That's why, we included a new author named "Md. Feroj Ahmed" was included in the author list to complete the revision successfully.

The contribution(s) of the author(s) being added or whose authorship status is changing are:

Formal Analysis, Methodology, Software, Validation, and Visualization

We confirm that the revised author list accurately reflects the contributions and responsibilities of the individuals listed as authors.

**Agreement and Consent**

By signing below, we confirm that:

1. We have reviewed and agreed to the proposed change in authorship described above.
2. We agree to the revised order and composition of the author list.
3. We confirm that the authorship change has been discussed among the authors and that there is no unresolved disagreement concerning the proposed change.
4. We agree that the revised author list may be submitted to **PLOS Neglected Tropical Diseases** for consideration and approval.
5. We understand that the final decision regarding the authorship change rests with PLOS.
6. We confirm that the information provided in this agreement is accurate and complete to the best of our knowledge.
7. We agree to the authorship and contribution information entered into the PLOS submission system.

**Signatures of All Authors for their agreements**

| **Author Name** | **E-Mail** | **Signature** |
| --- | --- | --- |
| **Sabkat Mahmud** | [sabkatmahmud@gmail.com](mailto:sabkatmahmud@gmail.com) |  |
| **Alvira Ajadee** | [alviraajadee@gmail.com](mailto:alviraajadee@gmail.com) |  |
| **Md. Ahad Ali** | [ahad.chembd@gmail.com](mailto:ahad.chembd@gmail.com) |  |
| **Md. Feroj Ahmed** | [ferojahmed.ru.stat178@gmail.com](mailto:ferojahmed.ru.stat178@gmail.com) |  |
| **Reaz Ahmmed** | [reazahmmed147@gmail.com](mailto:reazahmmed147@gmail.com) |  |
| **Md. Masud Rana** | [mmrana@bu.ac.bd](mailto:mmrana@bu.ac.bd); [mdmasud4129@gmail.com](mailto:mdmasud4129@gmail.com) |  |
| **Tasfia Noor** | [tasfianoor.cse.ruet@gmail.com](mailto:tasfianoor.cse.ruet@gmail.com) |  |
| **Md. Fahim Faysal** | [faysalfahim795@gmail.com](mailto:faysalfahim795@gmail.com) |  |
| **Md. Hadiul Kabir** | [hk@ru.ac.bd](mailto:hk@ru.ac.bd) |  |
| **Md. Nurul Haque Mollah** | [mollah.stat.bio@ru.ac.bd](mailto:mollah.stat.bio@ru.ac.bd) |  |

We respectfully submit this written agreement as documentation that all authors have provided their consent to the proposed change in authorship.

**On behalf of all authors,**

**Professor Md. Nurul Haque Mollah, PhD**

Corresponding Author
PLOS Neglected Tropical Diseases manuscript: PNTD-D-25-01313R2
